# Federated Learning for a Dynamic Edge: A Modular and Resilient Approach

**DOI:** 10.3390/s25123812

**Published:** 2025-06-18

**Authors:** Leonardo Almeida, Rafael Teixeira, Gabriele Baldoni, Mário Antunes, Rui L. Aguiar

**Affiliations:** 1 Intituto de Telecomunicações, 3810-193 Aveiro, Portugal; rafaelgteixeira@av.it.pt (R.T.); or mario.antunes@ua.pt (M.A.); or ruilaa@ua.pt (R.L.A.); 2 Departamento de Eletrónica, Telecomunicações e Informática, University of Aveiro, 3810-193 Aveiro, Portugal; 3 Gabriele Baldoni Consulting, 75000 Paris, France; gabriele.baldoni@gmail.com

**Keywords:** federated learning, resilient, fault tolerant, distributed machine learning

## Abstract

The increasing demand for distributed machine learning like Federated Learning (FL) in dynamic, resource-constrained edge environments, 5G/6G networks, and the proliferation of mobile and edge devices, presents significant challenges related to fault tolerance, elasticity, and communication efficiency. This paper addresses these issues by proposing a novel modular and resilient FL framework. In this context, resilience refers to the system’s ability to maintain operation and performance despite disruptions. The framework is built on decoupled modules handling core FL functionalities, allowing flexibility in integrating various algorithms, communication protocols, and resilience strategies. Results demonstrate the framework’s ability to integrate different communication protocols and FL paradigms, showing that protocol choice significantly impacts performance, particularly in high-volume communication scenarios, with Zenoh and MQTT exhibiting lower overhead than Kafka in tested configurations, and Zenoh emerging as the most efficient communication option. Additionally, the framework successfully maintained model training and achieved convergence even when simulating probabilistic worker failures, achieving a MCC of 0.9453.

## 1. Introduction

The fast growth of Edge Computing and Internet of Things (IoT) deployments, coupled with the generation of vast and often sensitive datasets at the edge, is driving demand for distributed machine learning paradigms such as Federated Learning (FL) [1]. These advancements are significantly enabled by the proliferation of edge devices, advanced network technologies like 5G and upcoming 6G providing high bandwidth and low latency, and the integration of Artificial Intelligence (AI) [2].

Applying AI to the vast, often sensitive, and inherently distributed datasets generated by these devices typically requires distributed processing paradigms that can handle the scale and characteristics of these environments, avoiding the need for massive data centralization [3].

FL has emerged as a particularly compelling distributed AI paradigm in this context, leveraging edge architectures to process IoT data while preserving data privacy and security. It allows multiple edge devices (e.g., IoT sensors, gateways, and mobile devices in an industrial or health setting), often communicating over these advanced 5G and 6G networks, to collaboratively train a shared global model without directly exchanging their raw, sensitive data [4]. To achieve this, instead of sharing the collected data, only model updates are communicated, addressing critical concerns around data ownership, regulatory compliance (such as the General Data Protection Regulation (GDPR) and AI Act [5]), and the need for low-latency, privacy-preserving processing at the edge.

However, deploying FL effectively in dynamic, resource-constrained, and potentially unreliable edge and IoT environments, despite the capabilities offered by networks like 5G and 6G, introduces significant practical challenges. These environments are characterized by frequent device disconnections, variable network conditions, heterogeneity in device capabilities, and the distributed nature of the data (often non-IID) [6]. Therefore, it is crucial to develop robust and resilient systems [7]. In this context, resilience refers to the ability of the system to maintain its training and performance, even in the presence of node failures, delays, dynamic network topologies, and other adversities.

In this paper, we propose a novel resilient federated learning framework, designed with modularity at its core, while highlighting the importance of resilience in FL and the critical role of communication protocols in achieving robust and scalable FL systems. This framework is built upon well-defined modules, each responsible for specific tasks within the FL process. By decoupling the various components of the system, we enable greater flexibility and adaptability, allowing for easy integration of new algorithms, communication protocols, and optimizations as they emerge in the rapidly evolving field of distributed deep learning.

The remainder of this document is organized as follows: First, we provide essential background information on distributed machine learning paradigms, clarify FL’s position among them, and evaluate the communication protocols critical for its distributed operation. Following this, we review related work on fault tolerance, elasticity, and security in existing FL systems. We then detail our proposed modular and resilient FL framework, describing its architecture and implementation. The methodology used for our experimental evaluation is then presented, covering the setup, dataset, and designed scenarios. Subsequently, we present and discuss the results of these experiments. Finally, the document concludes with a summary of our findings and outlines promising avenues for future research.

## 2. Background

Effective resilience mechanisms in FL can only be fully appreciated with a clear understanding of the underlying principles. Therefore, we present the necessary background before detailing our work on resilient FL. This includes exploring distributed Machine Learning (ML) paradigms, clarifying FL’s position among them, and evaluating the communication protocols critical for its distributed operation.

### 2.1. Distributed Machine Learning

Distributed ML operates by distributing the dataset or the model across multiple computational nodes, enabling parallel execution of the training phase. This approach can lead to faster training under certain conditions and is essential for handling models and datasets too large for a single machine [8,9]. The implementation of distributed training involves diverse architectures and techniques. A prominent taxonomy in [10] classifies these methods based on Centralized versus Decentralized Optimization, and Synchronous versus Asynchronous Scheduling.

**Figure 1:**
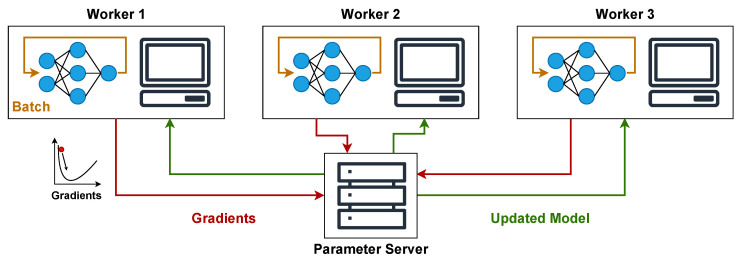
Architecture of a centralized optimization, illustrating the role of the Parameter Server in exchanging gradient updates with worker nodes after each batch.

Starting with centralized optimization, it employs a central component, often referred to as a parameter server. This server’s role is to receive gradient updates from distributed worker nodes, aggregate these updates, and then distribute the adjusted global model parameters back to the workers, as shown in Figure 1.

While this structure simplifies synchronization and overall management, the parameter server can become a bottleneck in larger deployments. This is due to the substantial communication load of sending gradients after each batch and receiving updated weights. This problem can be solved by distributing the parameter server across multiple nodes, although this introduces the need of synchronization and thus increases complexity.

**Figure 2:**
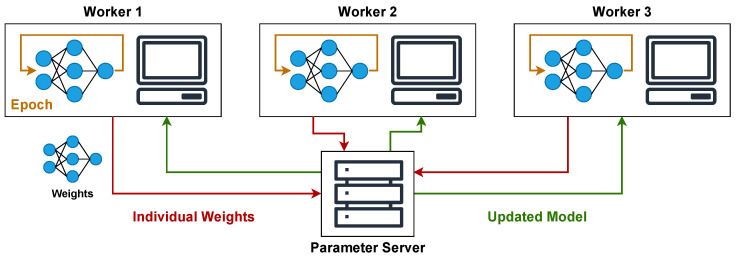
Decentralized optimization architecture with periodic synchronization, illustrating workers exchanging model weights with a Parameter Server per global communication round.

In decentralized optimization, the parameter server can also exist and be designated as the master node. Workers typically train their local models autonomously and synchronize periodically. This synchronization involves exchanging weights or model parameters directly with a subset of other workers or a designated master node within their group, as shown in Figure 2.

This method can improve scalability and resilience to communication latency or the failure of a single control point. Nevertheless, achieving consistent model convergence and minimizing performance drops across workers necessitates careful design and tuning of the synchronization protocols and hyperparameters. It’s also important to note that these centralized and decentralized strategies can be combined hierarchically. In such a hybrid model, workers might be organized into groups with a master node and a parameter server, which then aggregates updates from these group masters, which can further decrease communication overhead and enhance scalability [11].

Finally, distributed training systems manage the flow of computations through scheduling strategies, primarily classified as synchronous or asynchronous. In synchronous systems, all workers operate in lockstep, meaning that the system waits for all participating workers to complete their current task and report their updates before proceeding to the next iteration. This synchronized approach helps maintain consistency among workers and simplifies debugging. However, it can lead to inefficient resource utilization as faster workers remain idle while waiting for the slowest worker (the “straggler”).

Model aggregation in synchronous systems typically uses weighted averaging, where each worker’s update is scaled based on factors such as data size or importance before being combined into a global model.

The weighted averaging is defined as follows:

(1) $$\theta_{t+1}=\frac{\sum_{i=1}^{N}w_{i}\cdot \theta_{i}}{\sum_{i=1}^{N}w_{i}}$$

where $\theta_{t+1}$ is the updated model parameters, *N* is the number of workers, $w_{i}$ is the weight assigned to worker *i*, and $\theta_{i}$ is the model parameters from worker *i*. The weights are typically proportional to each worker’s data size or reliability. The normalization ensures that the update is a true weighted average, maintaining scale consistency and promoting a balanced aggregation.

On the other hand, asynchronous systems allow workers to complete their tasks and update the shared model independently without waiting for others. This maximizes resource utilization as workers are constantly processing. The challenge with asynchronous updates lies in managing potential inconsistencies introduced by updates based on stale model parameters, which can complicate convergence guarantees and sometimes degrade final model performance.

To mitigate this, linear interpolation is often used for model aggregation, blending new updates with the current model state to maintain stability.

The linear interpolation is defined as follows:

(2) $$\theta_{t+1}=\theta_{t}+\alpha \cdot \left(\theta_{i}-\theta_{t}\right)$$

where $\theta_{t+1}$ is the updated model parameters, $\theta_{t}$ is the current model parameters, α is the interpolation factor (0 < α < 1), and $\theta_{i}$ is the model parameters from worker *i*. This allows the system to blend new updates with the current model state, maintaining stability while incorporating fresh information.

While asynchronous systems are often more complex to implement and debug than synchronous ones, they can offer better efficiency in terms of resource utilization and higher scalability, especially in environments with heterogeneous worker speeds or unreliable connections.

### 2.2. Federated Learning

FL is a paradigm of distributed machine learning, set apart by key characteristics particularly relevant for privacy-sensitive and decentralized applications. It can be categorized into three main types based on how the data is distributed across participants [12]. Horizontal FL is applicable when the datasets held by different entities share the same feature space but differ in the samples they contain [13]. This is common, for example, with user data on mobile phones. On the other hand, Vertical FL is used when datasets share the same sample space but have different feature sets [14]. This might occur when different organizations hold complementary information about the same users. The third category, Federated Transfer Learning, is relevant when datasets differ in feature and sample space, leveraging transfer learning techniques to share knowledge between participants [15].

Due to its ability to handle decentralized and sensitive data without centralizing it, FL finds its most prevalent use cases in domains such as mobile computing, industrial engineering, and healthcare [16]. In these areas, data is often highly sensitive and restricted from being shared, or the sheer volume of data makes a traditional centralized approach impractical or impossible. Where these specific constraints on data sharing or centralization are absent, other distributed machine learning approaches might offer more suitable alternatives depending on the task and infrastructure.

### 2.3. Communication Protocols

Effective communication protocols are fundamental to the success of FL, playing a crucial role in managing the distributed training process efficiently while upholding privacy, ensuring scalability, and minimizing overhead. The selection of an appropriate protocol is highly dependent on specific system requirements, including the number of participating devices, network conditions, and security constraints. To understand the landscape of communication solutions for FL, we evaluated the scalability, fault tolerance, security features, and overall suitability of four selected protocols: Message Passing Interface (MPI) [17], Message Queuing Telemetry Transport (MQTT) [18], Kafka [19], and Zenoh [20], highlighting their respective advantages and drawbacks.

MPI is a well-established communication standard primarily utilized in High-Performance Computing (HPC) environments. It is designed for explicit data exchange via send and receive operations, enabling low-latency and high-throughput communication between nodes. However, its reliance on static configurations, requiring predefined communication endpoints, and a lack of built-in fault tolerance mechanisms make MPI less suitable for the dynamic and potentially unreliable nature of real-world FL systems where worker disconnections or failures are common. While its synchronous communication model ensures strong consistency, this strict synchronization can limit flexibility and hinder scalability in decentralized FL scenarios. Additionally, MPI does not provide native support for encryption or authentication, relying instead on secure underlying transport layers like Secure Shell (SSH) or network-level security configurations.

MQTT offers a lightweight, publish-subscribe messaging protocol particularly well-suited for resource-constrained devices and networks with limited bandwidth. It operates through a centralized broker that mediates communication, allowing clients to connect or disconnect dynamically without interrupting the system. It also offers a native way to notify clients when other clients disconnect, which is helpful for FL systems where worker availability may fluctuate. Despite its advantages for edge deployments, this centralized broker architecture can evolve into a bottleneck in large-scale fully decentralized FL systems as the volume of model updates needing exchange increases. MQTT leverages Transmission Control Protocol (TCP) and supports security features like Transport Layer Security (TLS), Mutual TLS (mTLS), client authentication, and role-based access control, but its fundamental dependence on a broker inherently limits its scalability compared to fully decentralized alternatives.

Kafka, a distributed streaming platform, is optimized for handling real-time data streams at scale. Its publish-subscribe model robustly supports dynamic and large systems, offering benefits such as persistent storage and strong fault tolerance through its distributed broker clusters. However, Kafka’s broker-based communication model introduces latency due to the additional hops required for routing messages. The only native disconnection notification mechanism is inspection of connected groups. To participate in a group, clients must subscribe with the same group ID, but this approach adds a considerable overhead because when a client joins or leaves, partitions must be rebalanced, which can be time-consuming and resource-intensive. This latency can impede the synchronization of model updates critical for the iterative training process in FL, potentially hindering its suitability for very low communication latency scenarios. Like MQTT, Kafka provides robust security options, including support for TLS, mTLS, client authentication, and role-based access control.

Zenoh is a more recent decentralized communication middleware designed explicitly for dynamic, resource-constrained, and potentially unreliable environments, addressing some limitations of broker-based systems. It adopts a fully decentralized architecture, removing single points of failure and facilitating seamless communication among devices even under challenging network conditions. Similar to MQTT, it offers a native way that allows participants to be notified when other participant leave the network. Zenoh is flexible, supporting multiple underlying communication methods such as TCP, offering TLS and mTLS for security, allowing adaptation to varying performance and security needs. Zenoh also supports the use of Access Control Lists (ACLs), enabling fine-grained permission management to control which entities can publish or subscribe to specific data. Despite its promising features for decentralized and resilient communication, Zenoh is still a relatively new protocol with a less mature ecosystem compared to more established solutions like MPI, MQTT, and Kafka, which might affect its immediate widespread adoption in existing FL deployments.

## 3. Related Work

While FL offers significant privacy advantages for distributed ML, deploying robust and efficient FL systems presents several practical challenges that include ensuring continuous operation despite failures, adapting to dynamic device participation, scaling to potentially vast numbers of clients, and securing communications.

A primary concern is Fault Tolerance, the ability to maintain system operation even when client devices disconnect unexpectedly or fail during training rounds. Various strategies have been proposed, ranging from architectural modifications like hierarchical aggregation using serverless functions [21], distributed aggregation centers [22], or leveraging consensus protocols such as Raft for aggregator failover and state replication [23], to protocol-level mechanisms including redundancy checks and task reassignment [24]. Additionally, some works explore the potential of robust communication backbones, like publish-subscribe systems (e.g., Apache Kafka), as conceptually discussed in [25], though not yet implemented. Many secure aggregation schemes inherently tolerate client dropouts, contributing to fault resilience [26,27].

Closely related is Elasticity, which addresses the dynamic nature of edge environments where devices may join or leave the training process at any time. Some approaches explicitly design algorithms and architectures using actor-based or adaptive aggregation strategies to handle asynchronous participation and variations in the set of collaborating devices [28].

Scalability is essential for supporting large numbers of participating devices. Architectural innovations, such as leveraging serverless aggregation topologies [21] or distributed networks of aggregation centers [22], aim to overcome the bottleneck of a single central server and reduce communication overhead on the server. The scale considered in evaluations varies across studies, offering different perspectives on practical deployment limits.

Finally, Security is foundational to FL’s promise. Beyond preserving data locality, protecting model updates during transit and aggregation is critical. Techniques include integrating lightweight cryptography and data compression [24], and employing advanced cryptographic primitives such as homomorphic encryption and secret sharing [26,27]. These schemes ensure the server cannot inspect individual updates and often allow model aggregation even in the presence of client dropouts.

Table 1 provides a comparative overview of these related works across key dimensions, including their specific approaches to Fault Tolerance and Elasticity, the scale of their evaluations, security mechanisms employed, evaluation methodologies (use of standard datasets and benchmarks to evaluate the performance of the solution), and code availability (open-source and reproducibility of results).

The Compliance column represents the overall alignment of each related work with the desired characteristics for a robust and scalable FL framework, particularly those relevant to dynamic and resource-constrained edge environments. This percentage reflects how comprehensively each study addresses the various dimensions presented in the table, with each dimension considered to have equal importance/weight in the context of our proposed system’s objectives.

The results reveal that while Fault Tolerance mechanisms are universally incorporated, Elasticity for dynamic networks is less common (present in two of eight systems). Security approaches range from lightweight stream ciphers to advanced cryptography like homomorphic encryption and Shamir’s secret sharing. Regarding evaluation methodologies, the predominant use of standard datasets (e.g., MNIST, CIFAR-10, FashionMNIST) may limit relevance for real-world IoT or Intrusion Detection System (IDS) scenarios where sensitive data protection is paramount. Finally, limited code availability (three of eight) hinders reproducibility and community progress.

## 4. Proposed Solution

Communication protocols hold a critical role in enabling efficient, secure, and scalable FL systems [29]. As we have discussed in the previous sections, each specific protocol has unique strengths and limitations. This makes each one suitable for different kinds of scenarios. Therefore, the protocol selection must be based specifically on the system’s requirements. In our case, a centralized server (parameter server) coordinates the training process, and the system is designed to be resilient to worker disconnections. This means that a centralized communication broker such as MQTT is not a problem, and the system can benefit from its lightweight nature. However, MPI is unsuitable for our system because it is designed for static configurations. Table 2 summarizes the communication protocols’ main features, highlighting their suitability for our scenario.

Understanding these protocol characteristics is essential for designing resilient and scalable FL systems capable of effectively handling practical challenges, including fault tolerance and dynamic participation, as reviewed in the following section. Building on these insights, we propose a novel, modular, and resilient Federated Learning framework designed to operate effectively in dynamic and potentially unreliable edge environments. This framework aims to advance the addressing of key challenges such as fault tolerance, elasticity, and communication flexibility by decoupling core functionalities into distinct modules and incorporating specific resilience mechanisms tailored to the unique characteristics of edge devices and networks.

### 4.1. Architecture and Overview

**Figure 3:**
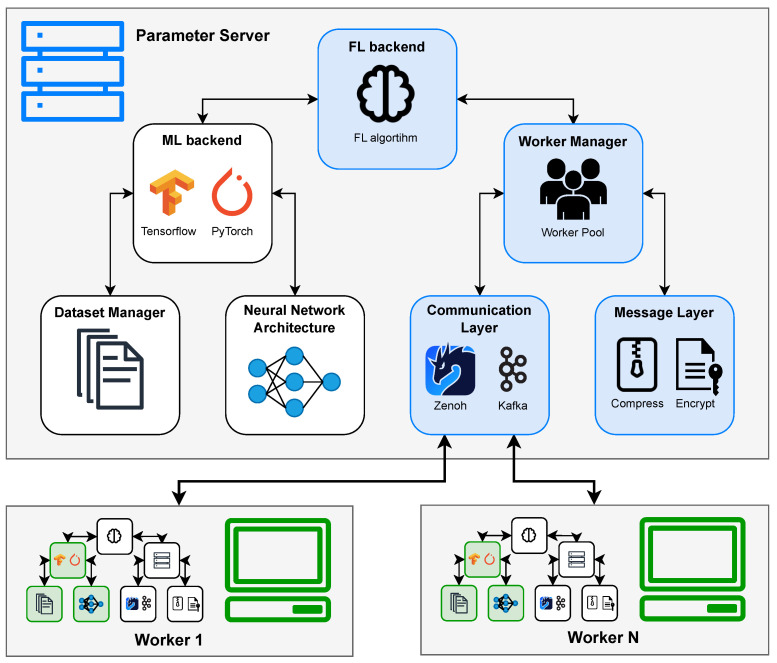
Architecture of the proposed modular and resilient Federated Learning framework.

Our proposed framework is not a single, rigid Federated Learning implementation, but rather a flexible architecture comprising seven core modules that work together to enable a variety of FL algorithms and configurations. This modular design is central to the framework’s adaptability, allowing for easy integration of new algorithms, communication protocols, resilience strategies, and optimizations without requiring extensive changes to the entire system. Figure 3 illustrates the architecture of our proposed framework and Table 3 provides a summary of the core modules and their functionalities. The seven core modules are as follows:

FL Backend: This module is the heart of the Federated Learning process logic. On the Parameter Server, it manages the global training loop, coordinates rounds and defines the workers scheduling, aggregates model updates from workers, and updates the global model state and validates it. On the worker side, depending on the algorithm, it manages the local training loop and interaction with the server or peers. It uses the ML Backend to interact with the model and the Worker Manager to interact with the workers.

ML Backend: This module provides an abstraction layer for the underlying machine learning framework (e.g., TensorFlow 2.18, PyTorch 2.6), making the rest of the framework agnostic to the specific library used. It manages the machine learning model, providing interfaces for common operations such as Save/Load Model, Train the model on local data, Predict using the model, and other general Model Operations like accessing weights or gradients. It uses the Dataset Manager to extract the data and the Neural Network Architecture to define the model.

Dataset Manager: Responsible for all aspects related to data handling on the client devices. It manages the data extraction process from local storage, potentially handling tasks like Download (if data needs to be fetched), Preprocess the data, and Load split data batches for training, validation, and testing. It provides processed data samples to the ML Backend.

Neural Network Architecture: This module defines the structure of the ML model being trained. It contains the specifications for the layers and connections of the neural network and is used by the ML Backend to create the model instance before the training begins. Separating this allows for easy swapping of model architectures for the same dataset or FL scenario.

Worker Manager: Primarily residing on the Parameter Server, this module manages the pool of participating client devices (workers). It keeps track of connected workers, handles joins/disconnects from the network, and manages the Worker subpool used for each training round (especially in client-selection scenarios), making it the core component to ensure the system’s Elasticity and Fault Tolerance. It uses the Communication Layer and the Message Layer to interact with the workers.

Communication Layer: This critical module handles all network communication between any nodes in the system, including the server and workers. It provides an abstract Send and Receive interface, hiding the complexities of the specific communication protocol used. Crucially, this layer is also responsible for providing foundational Resilience to the system by managing connection state, handling message delivery issues, and integrating protocol-specific fault tolerance features.

Message Layer: Positioned below the Communication Layer conceptually, this module is responsible for defining the format and handling the content of messages exchanged. It includes functionalities to Encode and Decode messages, and can optionally perform data Compression and Encryption of model updates or other exchanged information to enhance efficiency and security.

### 4.2. Implementation

We have implemented our modular and resilient Federated Learning framework in Python 3.12 to validate the design and demonstrate its feasibility.

The FL Backend module was implemented to provide variations of four key distributed training paradigms discussed in Section 2.1: Centralized Synchronous, Centralized Asynchronous, Decentralized Synchronous, and Decentralized Asynchronous Federated Learning. These implementations incorporate specific design choices and optimizations to improve performance and resilience.

The ML Backend module was developed with support for multiple popular deep learning libraries, including TensorFlow, PyTorch, and Keras (with support for Jax). This abstraction allows users to define their models using their preferred framework without requiring changes to other parts of the FL framework. The Neural Network Architecture module interfaces with the ML Backend to define the specific model structure for a given experiment.

For the Communication Layer, we implemented interfaces for the four distinct protocols analyzed in Section 2.3: MPI, Zenoh, MQTT, and Kafka. The implementation details for handling dynamic environments and failures vary significantly based on the chosen protocol. MPI, designed for static HPC environments, does not inherently support dynamic worker joins or failures gracefully within the framework’s communication handling. In contrast, the implementations for Zenoh and MQTT leverage their native support for asynchronous message handling via threading and built-in capabilities to detect client disconnections (e.g., through callbacks), enabling responsive fault detection from the parameter server’s perspective. Kafka, lacking native real-time disconnection notifications without incurring partition rebalancing overhead, required manual implementation of fault detection mechanisms using heartbeats and threading to manage incoming messages asynchronously.

Worker scheduling is handled with a default Round Robin strategy for selecting a subpool of clients for each training round. The size of this subpool is configurable, contributing to the system’s scalability and allowing operation even when only a subset of potential workers is available. The framework allows workers to join and leave the training process dynamically. How these events impact the training round depends on the chosen FL algorithm implementation:

- In synchronous approaches, an epoch or global communication round is considered complete when a pre-defined threshold (e.g., 50% of the selected subpool) of workers successfully submit their updates. This allows training to continue despite the failure or disconnection of a minority of workers.
- In asynchronous approaches, if a worker exits during a training task, the task is automatically reassigned to the next available worker according to the scheduling policy, ensuring that necessary computations are eventually completed.

Additional optimizations were implemented across FL algorithm types. For synchronous approaches, weighted averaging of model updates (gradients or weights) is performed based on the number of local samples each worker uses. The implemented framework is available as a Python package named flexfl on PyPI, complete with Command Line Interface (CLI) tools. This utility facilitates the execution of complex FL experiments by allowing users to specify the desired configuration, including the implementations for each of the seven modules and their specific arguments, by command line and via a configuration file. Addressing the challenge of managing a wide array of potential options and avoiding library conflicts (e.g., between TensorFlow and PyTorch).

## 5. Experimental Methodology

To validate the proposed modular and resilient Federated Learning framework and evaluate its performance and robustness in simulated edge environments, we designed and conducted a series of experiments. The experimental setup was meticulously crafted to emulate scenarios involving resource-constrained client devices participating in a federated training process coordinated by a more powerful central server.

### 5.1. Experimental Setup

The experimental environment was deployed on an ARM-based server infrastructure. This setup consisted of 11 Virtual Machines (VMs) running Ubuntu 24.04 LTS. One VM was designated as the parameter server, responsible for coordinating the federated learning rounds, aggregating model updates, and managing worker participation. This server VM was provisioned with 8 CPU cores and 16 GB of RAM and was configured with the maximum bandwidth of approximately 5 GB. This configuration ensured that the parameter server could handle the computational load of aggregation and avoid becoming a network bottleneck, allowing us to focus on communication challenges between the server and the workers.

The remaining 10 VMs were configured as worker nodes, simulating resource-constrained edge devices. Each worker VM was provisioned with 2 CPU cores and 4 GB of RAM. To further replicate realistic distributed network conditions often encountered by edge devices, a network bandwidth cap of 50 Mbps was imposed on each worker VM.

However, accurately measuring communication times and synchronization events is crucial for evaluating the framework’s performance and resilience. To achieve this, precise clock synchronization across all participating nodes was essential. The parameter server VMs was configured to function as an Network Time Protocol (NTP) server, and all worker nodes were synchronized with it. This setup minimized time drift between the machines, enabling reliable calculation of communication latencies and event timestamps throughout the experiments.

### 5.2. Dataset and Model

IDS and IoT environments stand out as highly practical and pertinent domains for FL implementation, primarily due to the critical need for data privacy and security, alongside the inherent distributed nature of data sources in these settings.

While well-established datasets like KDD98 [30], KDDCUP99 [31], or NSLKDD [32] exist for training IDS models, they are widely regarded as outdated and not fully representative of today’s network traffic, as detailed in [33]. This significantly restricts their applicability to contemporary network security research. Considering this limitation, we decided to use two recent and widely-recognized state-of-the-art dataset, UNSW-NB15 [34].

This dataset, developed by the Australian Centre for Cyber Security (ACCS), is designed for network intrusion detection and combines real modern normal traffic with synthetically generated attack traffic across nine distinct attack categories. The dataset comprises over 2 million records with 49 features. For this study, we used a preprocessed version featuring NetFlow characteristics (e.g., total bytes transferred, number of packets, duration), as published in [35] by the University of Queensland. We focused on the binary classification task, aiming to classify network traffic as either normal or an attack.

The dataset was partitioned into three main sets: 70% for training, 15% for validation, and 15% for future testing. The training data was distributed among the 10 worker nodes, with each worker receiving approximately 7% of the total dataset. A critical aspect of this data handling, consistent with the principles of FL, is that each worker node performed data normalization using only the statistics (e.g., mean and standard deviation) derived from its own local data split, without sharing raw data or statistics with other nodes or the server.

In this FL setup, the validation set was used by the parameter server for periodic evaluation of the global model’s performance throughout the training process. It is important to note that, for our experiments, the validation data was not used to influence the aggregation mechanism or global model updates, thereby ensuring the reported metrics effectively represent the model’s generalization performance on unseen data, akin to a test set. Furthermore, the resilience mechanisms integrated into the system, such as handling client dropouts, do not alter the aggregation logic or influence the model update process. This ensures consistency and integrity in the training dynamics. Nonetheless, the modular design of the FL Backend does allow for future integration of validation-aware aggregation strategies.

Based on a prior evaluation of model types on the UNSW-NB15 dataset conducted in [36] which compared Artificial Neural Network (ANN), Convolutional Neural Network (CNN), and Recurrent Neural Network (RNN), we selected the ANN model for our experiments. This choice was driven by the ANN’s relative simplicity and faster training time while still demonstrating performance comparable to the more complex models on this dataset. The chosen ANN architecture consists of an input layer followed by three hidden layers with 128, 96, and 64 neurons, respectively. A dropout layer with a rate of 0.25 was included after the hidden layers for regularization. The ReLU activation function was applied to all hidden layers, softmax to the output layer, and the model was trained using the Adam optimizer.

### 5.3. Experimental Scenarios

We designed two main experimental scenarios to evaluate different facets of the framework’s performance and resilience. The first scenario compares the communication overhead of various protocols implemented and compares different FL approaches. The second scenario investigates the framework’s ability to handle worker failures and dynamic participation under multiple configurations, demonstrating its robustness and the continued convergence of the trained model. The setup, execution, and analysis of these scenarios are detailed in the following section.

Both scenarios were executed in a controlled environment, ensuring that all variables except those tested remained constant. These core hyperparameters, which are fully tunable within the FlexFL framework to adapt to diverse FL scenarios and requirements, are summarized in Table 4.

## 6. Results

This section presents the results of the two experimental scenarios designed to evaluate the proposed framework. These experiments demonstrate the framework’s flexibility in integrating different communication protocols and its resilience to worker failures while ensuring effective model training.

### 6.1. Scenario 1: Impact of Communication Protocols and FL Algorithms on System Performance

This scenario evaluated the communication overhead of Zenoh, MQTT, and Kafka when used as the underlying communication protocol in our Federated Learning framework. MPI is not evaluated because of its limitations in dynamic scenarios. We conducted experiments using different FL approaches (Decentralized sync/async and Centralized sync) to simulate varying communication patterns and loads, allowing for a comparison of the protocols and FL approaches.

As discussed in Section 2, these approaches differ significantly in how and when model updates are communicated between workers and the server, leading to distinct communication loads. Decentralized approaches with epoch-based updates generally incur lower per-epoch communication volume than the Centralized Synchronous approach, which updates per batch. Additionally, synchronous approaches are expected to be slower than asynchronous ones, as they require all workers to synchronize before proceeding to the next round.

Table 5 summarizes the results of the experiments, showing the workers’ average communication and working time over three runs for each protocol and FL approach. As we can see, Zenoh consistently demonstrates the lowest average communication time across all tested FL approaches, with MQTT being closely competitive. In contrast, Kafka exhibits a significantly higher average communication time.

This difference is particularly evident in the Centralized Sync approach, which involves a much higher frequency and volume of communication compared to the decentralized methods. For instance, Centralized Sync averaged around 2285 messages sent per worker with an average total payload of approximately 208 MB per worker throughout the run. In contrast, decentralized methods averaged only about 17 messages per worker with a much smaller average total payload of approximately 1.4 MB per worker. This makes the communication protocols with higher latency more impactful in the Centralized Sync setting.

The average working time remained relatively similar across all protocols within the same FL approach, which indicates that the communication protocol does not significantly affect the workers’ computation time. However, Kafka shows slightly higher working times, which could be partially attributed to the manual implementation of asynchronous message reception and fault detection using Python threading in our framework, due to the lack of native support, potentially faces limitations from Python’s Global Interpreter Lock (GIL) [37] compared to protocols with more optimized native asynchronous handling or implementations in languages without such constraints.

When analyzing MQTT in the Decentralized approaches, we observe that the communication time is slightly higher in the synchronous setting compared to the asynchronous one. This suggests that the simultaneous arrival of synchronous updates from multiple workers might put additional strain on the central MQTT broker, leading to increased processing or queuing delays reflected in the communication time.

Comparing the FL approaches, Centralized Sync using Zenoh or MQTT results in a lower Average Total Run Time than Decentralized Sync. However, observing the Average Working Time reveals a crucial difference. Workers in the Centralized Sync approach spend significantly less time on local computation compared to Decentralized Sync workers. This indicates that while the overall time is lower (due to the powerful parameter server handling aggregation), the parameter server in Centralized Sync can act as a bottleneck, with workers spending considerable time waiting for the server to aggregate updates and distribute the new model.

As expected based on the design principles discussed in Section 2, the Decentralized Async approach consistently achieves a lower Average Total Run Time compared to Decentralized Sync across all protocols. This is because asynchronous workers do not wait for all participants to complete their local tasks before proceeding to the next update cycle, thereby mitigating the straggler problem inherent in synchronous methods and improving overall system utilization.

**Figure 4:**
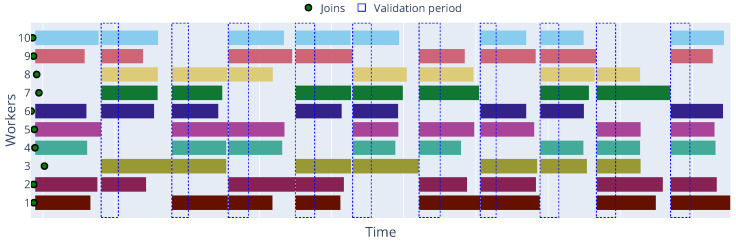
Timeline illustrating worker activity and idle periods across training global communication rounds in the Decentralized Synchronous FL approach with Zenoh, showing the waiting for synchronization points.

**Figure 5:**
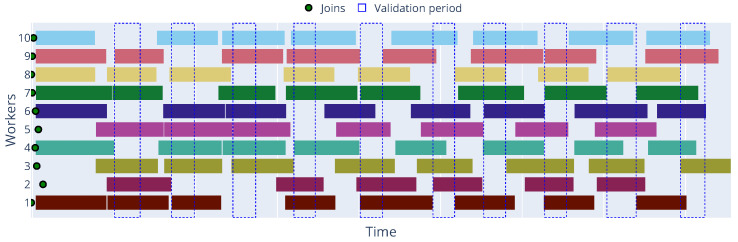
Timeline illustrating worker activity and idle periods across training global communication rounds in the Decentralized Asynchronous FL approach with Zenoh, demonstrating independent task completion and update submission.

Figure 4 and Figure 5 illustrate the timeline of the Decentralized Synchronous and Asynchronous approaches, respectively, where we can observe, in the first case, the parameter server waits for all workers to finish their local training before aggregating the updates, while in the second case, the workers can send their updates to the parameter server as soon as they finish their local training, allowing for a more efficient use of resources.

This inherent advantage of asynchronous FL is particularly critical in dynamic edge environments characterized by significant device heterogeneity, where worker speeds can vary drastically. When a worker joins, metadata is shared with the Parameter Server, this metadata can include information regarding their computational capabilities (e.g., CPU, memory, or GPU availability) and current network conditions (e.g., bandwidth and latency) and can be used in our modular Framework to implement more sophisticated scheduling policies to further improve performance.

### 6.2. Scenario 2: Fault Tolerance Evaluation with Worker Failures

This scenario tested the framework’s ability to maintain the training process and achieve model convergence in the presence of worker failures. We focused on the Decentralized Asynchronous FL configuration, which is well-suited for dynamic environments and often more inherently tolerant of delays and dropped participants compared to strict synchronous methods. Experiments were conducted with Zenoh, MQTT, and Kafka as communication protocols, introducing probabilistic worker failures at different rates. Workers were simulated to perform a graceful exit and attempt to rejoin the training process shortly after a failure event. Failure events occurred every 10 s with probabilities of 1% and 5% for each worker being selected for a potential failure event during that interval.

While experiments were performed using all three protocols, the results for Zenoh are presented here as a representative example, showing the framework’s resilience to worker disruptions across varying failure rates within the Decentralized Asynchronous setting. Results for MQTT and Kafka showed similar trends in maintaining training progress and convergence under this setup and are available for review in GitHub at https://github.com/leoalmPT/FlexFL/tree/MDPI (acessed on 15 June 2025).

To quantify the impact of failures and evaluate the fault tolerance mechanisms, we tracked the state of each worker within each training epoch or global communication round. A worker’s status during a round can be categorized into one of five states:

(i) Idle without fail: The worker was available and did not experience a failure event while idle waiting for a task or the next round.
(ii) Worked successfully without failing: The worker was assigned a local training task, completed it, and successfully communicated its update to the server without experiencing a failure event during this epoch.
(iii) Failed while idle: A failure event occurred while the worker was available but not actively performing a local training task or communicating. This is generally less disruptive as no computation result is lost. This is considered a non-critical failure as the worker can rejoin the training process without any loss of contribution.
(iv) Failed while working: A failure occurred while the worker actively performed a local training task or communicated their update. This is considered a critical failure as the ongoing computation or communication is interrupted, potentially requiring task re-assignment in an asynchronous setting.
(v) Failed after working successfully: The worker completed its local training task and successfully communicated its update, but experienced a failure event afterward, before the start of the next round. This is considered a non-critical failure as the contribution for the current round was successfully made.

Table 6 summarizes the occurrence of critical and non-critical failures across the three communication protocols at both 1% and 5% failure rates. The table shows the total number of failures observed across all workers and epochs and classifies them into critical and non-critical categories.

The results in Table 6 indicate that while failures (both critical and non-critical) increase with a higher failure rate as expected, the framework’s design, particularly the Decentralized Asynchronous approach coupled with the communication layer’s handling of disconnections, allows the training process to continue. Although the runs were performed with the same seed, to ensure consistent failure events across all protocols, the results vary in different runs due to the total duration of the run and the workers’ performance (e.g., different working times might lead to other workers being selected for training tasks).

**Figure 6:**
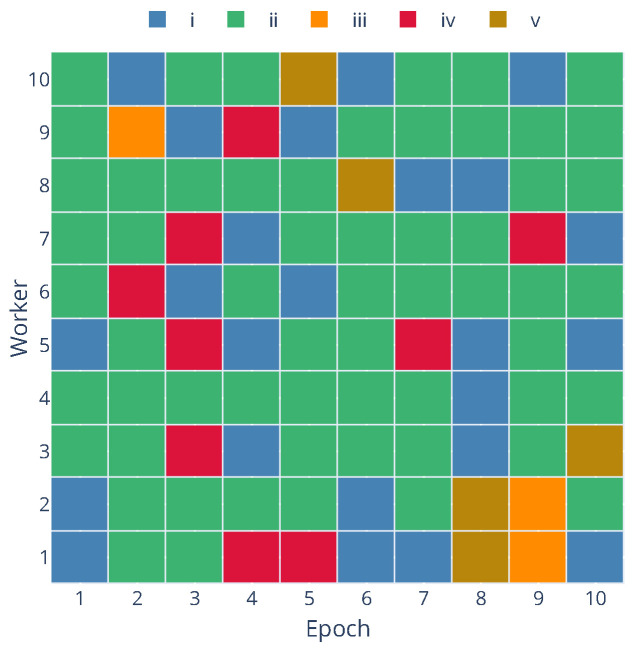
State matrix of workers during training with Zenoh protocol at 5% failure rate.

**Figure 7:**
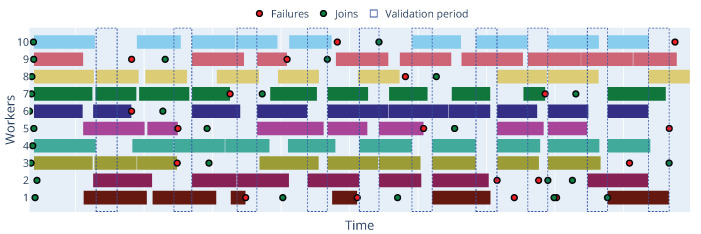
Timeline of workers during training with Zenoh protocol at 5% failure rate.

To provide a deeper insight into the dynamic behavior of workers and the occurrence of different states over time, Figure 6 presents a state matrix visualization for each worker across the epochs for the Zenoh protocol with a 5% failure rate and Figure 7 shows the timeline of the same experiment. In this timeline, we can observe each worker’s working and idle periods, as well as the occurrence of failure events. Additionally, the timeline shows how the parameter server handles worker failures and reassigns tasks to available workers. By default, the parameter server will only process updates and worker disconnections outside the validation period, and it will pause the training process if the minimum number of workers (workers per epoch) is not reached.

**Figure 8:**
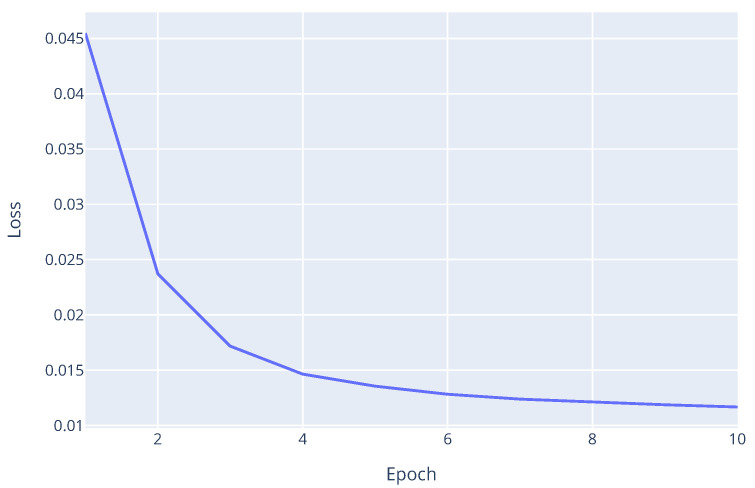
Validation loss curve during training with Zenoh protocol at 5% failure rate.

Most importantly, despite the introduced failures and the dynamic nature of the worker pool, the framework was able to train the model to converge successfully. Figure 8 shows the validation loss curve over epochs for the Zenoh protocol with a 5% failure rate.

The decreasing trend in the loss plot indicates that the model continued to learn and converge effectively, even under the stress of a 5% probabilistic worker failure rate. This demonstrates the robustness of the proposed framework’s fault tolerance mechanisms within the Decentralized Asynchronous FL setting. Final evaluation metrics on the validation dataset are similar across all experiments, with a final Matthews Correlation Coefficient (MCC) of 0.9453, f1-score of 0.9957, and accuracy of 0.9956 for this last experiment. This confirms that the converged model achieved performance comparable to training without failures, proving its practical utility in unreliable edge environments.

For this evaluation, we primarily focused on global model validation metrics to assess the framework’s overall convergence and generalization capabilities on unseen data, rather than tracking training metrics from individual clients. This choice was made to avoid additional overhead, and more importantly, because local training metrics in a federated setting, especially under non-IID data distributions, can be misleading and do not reliably reflect the global model’s learning progress. In such scenarios, client-specific metrics often exhibit high variance and are not indicative of the model’s ability to generalize, making global validation performance a more meaningful and consistent measure.

## 7. Conclusions

The fast growth of AI and the increasing availability of vast datasets have propelled distributed ML, particularly FL, to the forefront of modern computing. However, employing FL in dynamic, resource-constrained, and potentially unreliable edge environments presents significant fault tolerance, elasticity, and communication efficiency challenges. This paper addressed these critical issues by proposing and implementing a novel modular and resilient FL framework designed to operate effectively in such challenging settings.

Through a series of experimental evaluations conducted in simulated edge environments, we validated the efficacy and robustness of the proposed framework. The first scenario demonstrated the framework’s flexibility in integrating various communication protocols (Zenoh, MQTT, and Kafka) and FL approaches (Centralized Synchronous, Decentralized Synchronous, and Decentralized Asynchronous). We observed that the choice of communication protocol significantly impacts performance, especially in scenarios involving high message volumes characteristic of synchronous and centralized methods.

The second scenario evaluated the framework’s resilience to worker failures within a Decentralized Asynchronous configuration. Despite introducing probabilistic worker failures, the framework successfully maintained the training process and achieved model convergence with performance metrics on the validation dataset comparable to training runs without simulated failures. This experimental evidence strongly supports the framework’s ability to handle dynamic worker participation and withstand disruptions common in edge deployments. Notably, Zenoh emerged as the most efficient communication protocol.

### 7.1. Limitations

While the proposed modular framework and the conducted experimental evaluation demonstrate promising capabilities in terms of flexibility and resilience, it is important to acknowledge certain limitations inherent in the current study and experimental setup. Although designed to emulate resource-constrained edge devices and network conditions, the simulated environment was subject to specific constraints that may not fully represent the complexity and variability of real-world deployments.

For example, the worker nodes were implemented as Virtual Machines (VMs) with relatively uniform hardware specifications (CPU, memory) and were allocated dataset partitions that approximated an Independent and Identically Distributed (IID) data scenario. Real-world edge environments typically exhibit significant heterogeneity in device capabilities and widely divergent, non-IID data distributions, which can introduce additional complexities and challenges for achieving robust model convergence and consistent performance [38,39]. These VMs also simulated worker failures with graceful exits followed by potential re-joins. This controlled simulation does not fully capture the unpredictable nature of abrupt, unhandled crashes, sudden power loss, or prolonged network disconnections that can occur in practice.

Additionally, the scale of the experimental evaluation, involving a relatively small number of worker VM (10), provides a valuable insight into the framework’s behavior and easier visualization of results, but does not definitively validate its scalability to massive FL deployments involving potentially hundreds of devices.

### 7.2. Future Work

Several promising directions for future work can be pursued, building upon the foundation established by this research and aiming to address the identified limitations.

A primary direction is to significantly expand the scope of the experimental validation to encompass a wider variety of challenging real-world scenarios. This includes evaluating the framework with diverse and larger datasets from different domains (such as healthcare, finance, and industrial IoT), particularly under non-IID data distributions, and deploying the framework on heterogeneous hardware platforms to assess and optimize its performance under varying computational resources comprehensively.

The framework’s modular design offers a key advantage in addressing challenges posed by non-IID data distributions, a prevalent characteristic of real-world edge environments [38]. Specifically, the FL Backend module, which orchestrates the global training loop and aggregates model updates, is ideally positioned to integrate advanced aggregation strategies designed for non-IID data and integrate worker scheduling with intelligent client selection mechanisms that prioritize data diversity or balance contributions from heterogeneous data distributions in each round. The Dataset Manager module, responsible for local data processing on client devices, could be extended to incorporate local data augmentation, re-sampling techniques, or even privacy-preserving methods to expose local data statistics that the FL Backend could use for more informed aggregation, and for a improved scheduling policy.

A critical direction for future work is the comprehensive evaluation of the framework’s expected scalability to a much larger quantity of edge devices, ranging from hundreds to thousands. Such an expansion highlights potential bottlenecks, predominantly the central parameter server and the communication broker’s capacity. To handle this challenge, we can integrate advanced distributed aggregation strategies such as hierarchical aggregation [11], where multiple intermediate aggregators handle subsets of devices before a global server consolidates their updates.

Furthermore, a detailed investigation into the effect of integrating various data compression mechanisms within the Message Layers is needed. While the framework includes optional compression capabilities, a performance analysis quantifying the impact of different compression algorithms on communication overhead and overall training time is also necessary.

Moreover, given the critical importance of privacy and security in FL, particularly in sensitive edge environments, the Message Layer’s optional encryption capabilities also needs to be addressed to understanding the performance overhead for practical deployments. It is important to note that many modern edge devices are increasingly equipped with hardware-accelerated cryptographic modules or dedicated processor instructions that can significantly reduce the computational burden of standard symmetric encryption schemes, making the performance impact for basic encryption quite minimal or negligible in many practical scenarios, thereby ensuring privacy without significantly impeding the training process [40,41].

## Figures and Tables

**Table 1:** Overview of related works.

Ref	Fault Tolerance	Elasticity	Scalability	Security	Evaluation	Code	Compliance
[25]	🗸	×	Not tested	🗸	×	×	33%
[24]	🗸	×	1000	🗸	🗸	×	66%
[21]	🗸	🗸	10,000	×	🗸	×	66%
[26]	🗸	×	400	🗸	×	×	50%
[27]	🗸	×	1000	🗸	×	🗸	66%
[22]	🗸	×	600	×	🗸	×	50%
[28]	🗸	🗸	64	×	🗸	🗸	83%
[23]	🗸	×	10	×	🗸	🗸	66%
Total:	100%	25%	87.5%	50%	62.5%	37.5%	

**Table 2:** Summary of communication protocols for Federated Learning.

Protocol	Scalability	Fault Tolerance	Security	Suitability
MPI	Limited	No built-in support	Relies on SSH	Low, lacks fault tolerance and scalability
MQTT	Moderate	Moderate	TLS, mTLS, role-based	High, suitable when using a central broker
Kafka	High	High	TLS, mTLS, role-based	Moderate, but latency can hinder synchronization
Zenoh	High	High	TLS	High, but limited ecosystem maturity

**Table 3:** Core modules of the proposed framework and their functionalities.

Module	Functionality
FL Backend	Master and worker training loops, model aggregation, and validation.
ML Backend	Save/Load model, train, predict and other model operations.
Dataset Manager	Download, preprocess, and load data.
Neural Network Architecture	Define the structure of the ML model.
Worker Manager	Handle messages, manage worker pool, and handle joins and disconnects.
Communication Layer	Send/receive messages, manage connection state, and handle delivery issues.
Message Layer	Encode/decode messages, optionally compress and encrypt data.

**Table 4:** Core hyperparameters used in the experiments, representing a typical configuration.

Hyperparameter	Value
ML Backend	Keras with TensorFlow
Batch size	1024
Learning rate	0.0001
Epochs or Global communication rounds	10
Local Epochs per worker	3
Worker threshold	50%
Workers per epoch	7
Loss function	Categotical Crossentropy

**Table 5:** Comparative results of different communication protocols and FL approaches.

FL Approach	Comm Protocol	Average Comm Time (s)	Average Working Time (s)	Average Total Run Time (s)
Decentralized Synchronous	Zenoh	0.7019	166.06	266.20
	MQTT	0.8745	165.44	272.66
	Kafka	2.0634	168.44	274.23
Decentralized Asynchronous	Zenoh	0.7043	169.47	240.59
	MQTT	0.7085	166.85	241.30
	Kafka	2.014	172.41	248.11
Centralized Synchronous	Zenoh	8.559	38.26	201.21
	MQTT	11.61	37.93	202.05
	Kafka	190.76	40.51	742.89

**Table 6:** Summary of worker failures during training with Decentralized Asynchronous approach.

Failure Rate	Comm Protocol	Non-Critical Failures	Critical Failures	Total Failures
1% every 10 s	Zenoh	2	1	3
	MQTT	2	1	3
	Kafka	1	2	3
5% every 10 s	Zenoh	8	9	17
	MQTT	7	9	16
	Kafka	5	11	16

## Data Availability

The original data presented in the study are openly available in Github at https://github.com/leoalmPT/FlexFL/tree/MDPI (accessed on 15 June 2025).
